# Community concerns about genetic discrimination in life insurance persist in Australia: A survey of consumers offered genetic testing

**DOI:** 10.1038/s41431-023-01373-1

**Published:** 2023-05-11

**Authors:** Jane Tiller, Andrew Bakshi, Grace Dowling, Louise Keogh, Aideen McInerney-Leo, Kristine Barlow-Stewart, Tiffany Boughtwood, Penny Gleeson, Martin B. Delatycki, Ingrid Winship, Margaret Otlowski, Paul Lacaze

**Affiliations:** 1 Public Health Genomics, School of Public Health and Preventive Medicine, Monash University, Melbourne, Australia; 2 Murdoch Children’s Research Institute, Parkville, Australia; 3 Australian Genomics, Melbourne, Australia; 4 Centre for Health Equity, Melbourne School of Population and Global Health, The University of Melbourne, Melbourne, Australia; 5 The University of Queensland Diamantina Institute, University of Queensland, Dermatology Research Centre, Brisbane, Australia; 6 Northern Clinical School, Faculty of Medicine and Health, University of Sydney, Sydney, Australia; 7 Deakin Law School, Melbourne, Australia; 8 Victorian Clinical Genetics Services, Parkville, Australia; 9 Department of Medicine, the University of Melbourne, Melbourne, Australia; 10 Genomic Medicine and Family Cancer Clinic, Royal Melbourne Hospital, Parkville, Australia; 11 Faculty of Law and Centre for Law and Genetics, University of Tasmania, Hobart, Australia

**Keywords:** Genetic counselling, Medical ethics, Genetics, Ethics, Clinical genetics

## Abstract

Fears of genetic discrimination in life insurance continue to deter some Australians from genetic testing. In July 2019, the life insurance industry introduced a partial, self-regulated moratorium restricting the use of genetic results in underwriting, applicable to policies up to certain limits (eg AUD$500,000 for death cover).

We administered an online survey to consumers who had taken, or been offered, clinical genetic testing for adult-onset conditions, to gather views and experiences about the moratorium and the use of genetic results in life insurance, including its regulation.

Most respondents (*n* = 367) had undertaken a genetic test (89%), and had a positive test result (76%; *n* = 243/321). Almost 30% (*n* = 94/326) reported testing after 1 July 2019. Relatively few respondents reported knowing about the moratorium (16%; *n* = 54/340) or that use of genetic results in life insurance underwriting is legal (17%; *n* = 60/348). Only 4% (*n* = 14/350) consider this practice should be allowed. Some respondents reported ongoing difficulties accessing life insurance products, even after the moratorium. Further, discrimination concerns continue to affect some consumers’ decision-making about having clinical testing and applying for life insurance products, despite the Moratorium being in place. Most respondents (88%; *n* = 298/340) support the introduction of legislation by the Australian government to regulate this issue.

Despite the introduction of a partial moratorium in Australia, fears of genetic discrimination persist, and continue to deter people from genetic testing. Consumers overwhelmingly consider life insurers should not be allowed to use genetic results in underwriting, and that federal legislation is required to regulate this area.

## Introduction

The use of genetic test results by life insurance companies in underwriting, and the associated impact on clinical and research outcomes, is a long-standing issue of international concern. Studies have described various ethical, medical and societal concerns with this practice, expressed by members of the public, consumers, and disease support groups [1–11].

In Australia, *private health insurance* is community-rated under the *Private Health Insurance Act 2007* (Cth), meaning genetic test results cannot be used by private health insurers to discriminate against applicants. However, for *life insurance* (including death, disability, trauma and income protection cover), an exception under section 46 of the *Disability Discrimination Act 1992* (Cth) (DDA) permits life insurance companies to use genetic test results in underwriting, if supported by actuarial data or “other relevant factors” on which it is reasonable to rely. Little judicial consideration has been given to the operation of s46 of the DDA, but the Federal Court of Australia has held (in a context outside of genetics) that “other relevant factors” can only be relied upon to justify discrimination if actuarial or statistical data is not available [12].

Research shows that insurance discrimination fears can deter individuals from having genetic testing [13, 14] and participating in genetic research [15]. Internationally, many countries have banned or restricted life insurance companies from using genetic test results in underwriting - to decline an application, restrict cover or increase the cost of premiums [16–18]. For example, the Canadian *Genetic Nondiscrimination Act* (2017) (GNA), prohibits the use of genetic test results in all insurance (among other services), and the US *Genetic Information Nondiscrimination Act* (2008) (GINA) bans the use of genetic test results in health insurance and employment contexts. The UK Code on Genetic Testing and Insurance [19], an agreement between the insurance industry and the UK Government introduced in 2001, bans the use of predictive genetic test results with a single exception - predictive genetic tests for Huntington disease, where the life insurance cover is > £500,000 (~ A$930,000).

In Australia, a Parliamentary Joint Committee hearing into the life insurance industry recommended a ban on this practice in 2018 [20]. The Australian government has not curtailed life insurers’ legal entitlement to use genetic test results under the DDA. However, in 2019 the life insurance industry body, the Financial Services Council (FSC) introduced a partial, self-regulated moratorium for applications up to certain limits, including AUD$500,000 for death and total permanent disability cover, $200,000 for trauma cover and AUD$4000/month for income protection [21]. The moratorium, which prohibits insurers from asking for and from using genetic results up to the prescribed limits, is not subject to any government oversight, and was set to expire in 2024 unless renewed. In October 2022, the FSC indicated that the moratorium would become indefinite when it is incorporated into the FSC Life Code (due to take place in July 2023).

Australian health professionals, involved in obtaining informed consent and explaining the implications of genetic testing to patients, have previously [22] reported concerns with life insurance discrimination related to genetic testing. These studies suggest some Australians are still declining or delaying clinical genetic testing, and some may not attend genetics clinics at all, due to fears about potential insurance discrimination. Despite the introduction of the FSC moratorium, health professionals remain concerned about the ongoing deterrent effect of genetic discrimination in Australia, and the lack of government regulation [23].

Genetic discrimination in insurance underwriting has had an impact on consumers internationally [24]. Historical experiences of discrimination reported by consumers include perceived coercion regarding genetic testing in order to obtain insurance [25]; unaffected relatives of individuals with genetic conditions reporting difficulty obtaining insurance, in some cases even with genetic results showing they do not have the familial pathogenic variant [26, 27]; and unaffected individuals with pathogenic variants whose risk-reducing measures are not considered [3, 28–31]. We previously surveyed Australian consumers, before the introduction of the FSC moratorium, to gauge their views about and experiences of genetic discrimination [32]. We found numerous instances of consumers reporting difficulties accessing life insurance products, including thirty-two individuals with no history of the relevant disease, who had undertaken risk-reducing measures.

The Australian Genetics and Life Insurance Moratorium: Monitoring the Effectiveness and Response (A-GLIMMER) study [33] was funded by the Australian Medical Research Future Fund Genomics Health Futures Mission in 2020 to monitor the effectiveness of the FSC moratorium by conducting research with four different stakeholder groups - consumers, health professionals, researchers and the financial services industry [24]. The present study was designed to ascertain updated views and experiences of Australian consumers who have had, or been offered, genetic testing for adult-onset conditions. The study was limited to adult-onset conditions because different considerations arise in the context of *predictive testing* of unaffected individuals for genetic risk of future disease, compared with *diagnostic testing* of individuals who already have symptoms or clinical diagnosis of disease. The FSC moratorium clearly indicates that it can use disease diagnoses (whether diagnosed through clinical or geniting testing) as a basis for discrimination, but that the moratorium applies to predictive genetic tests in applications below the financial limits. The moratorium protections do not apply to individuals with childhood-onset disease, who have already received a diagnosis by the time they apply for life insurance in adulthood.

## Methods

### Population and recruitment

The A-GLIMMER project protocol has been published previously [24]. This study was part of the consumer arm of the A-GLIMMER project, and its population of interest included Australians, over the age of 18, with or without life insurance products, who met the definition of either a “genetic tester”, “pre-tester” or “decliner”.

**Table:** 

***Genetic testers***	Individuals (affected or unaffected) who have already had a genetic test and received a genetic test result. This could be positive (unfavourable) or negative (favourable). Results may have been received prior to or following the introduction of the moratorium
***Pre-testers***	Individuals (affected or unaffected) who are eligible for and are actively considering having a genetic test
***Decliners***	Individuals who are eligible for but have chosen not to have a genetic test

Eligibility was established through screening questions at the beginning of the questionnaire, and defined as “Australians who have had, or are eligible for, a genetic test for a gene change that increases the chance of developing disease (either before or after developing symptoms of disease)”. This included predictive genetic testing, but excluded pre-conception carrier screening or prenatal testing. For the purposes of our study, respondents were included (considered eligible for a genetic test) if they had undertaken or been offered such a test, or their first-degree blood relative (sibling, parent or child) had undertaken such a test.

A range of targeted recruitment strategies were adopted to capture a broad sample, which included:

- Newsletters and email invitations to members of patient support and advocacy groups, including Lynch Syndrome Australia, Pink Hope, Mito Foundation, Breast Cancer Network Australia, Familial Hypercholesterolemia Network Australia, Australian Genetic Heart Disease Registry, Australian Genomics Consumer Advisory Group, and Rare Voices Australia;
- Social media advertisements;
- Newsletters emailed directly to members of the Human Genetics Society of Australasia (HGSA) and the Australian Genomics Health Alliance; and
- Snowball sampling.

Recruitment took place between October 2021 and February 2022. Following the online survey, respondents were invited to consent to future contact. Contact details were not collected if respondents preferred to remain anonymous.

### Survey development and data collection

We developed an online survey (see Supplementary Materials S1) using REDCap software [34].

The survey was adapted from our previous survey, that was administered before the introduction of the FSC moratorium [32]. The previous survey had been developed in partnership with consumer groups Lynch Syndrome Australia (LSA) and Pink Hope (PH). It was designed to collect data from respondents who had had genetic testing for genes associated only with Lynch syndrome or Hereditary Breast and Ovarian Cancer (HBOC). The current survey expands beyond this, collecting data from individuals *considering* having genetic testing (*pre-testers*), and who had decided not to have genetic testing (*decliners*), to help identify reasons for declining testing. Further, we broadened the survey’s scope to include testing for any gene change that increases the chance of developing disease. We also engaged a broader range of project partners (e.g., Rare Voices Australia, Australian Genomics, Familial Hypercholesterolaemia Network Australasia, Mito Foundation, Breast Cancer Network Australia, Rare Cancers Australia and the Australian Genetic Heart Disease Registry), in addition to LSA and PH. These groups helped design the new survey so it was relevant for a range of various conditions.

New questions were also introduced to assess levels of understanding of the moratorium, impact of the moratorium on decision-making and experiences with accessing life insurance products. Information about the terms of the moratorium was provided, before asking participants to rate key aspects as positive, neutral or negative (see Supplementary file S1, p14). We worked with a team of clinical and policy members to develop custom questions as validated scales were not available due to the recency of the FSC moratorium. Data were collected through closed-ended responses using nominal and fixed alternative options, with several open-ended questions where free text was allowed. The survey was piloted by clinicians and representatives from our partner consumer groups, and feedback used to refine survey wording.

### Data analysis

Quality control and descriptive analysis of the data were conducted using R 4.0.4 [35], with figures produced using ggplot2 [36]. Participants who provided their year of birth (optional) were divided into three age groups (18–39, 40–65, and 65 + ), to enable sub-group analysis for certain questions.

For some questions, respondents could use free text to provide further comments. Where applicable, these free-text fields have been categorised and reported, to provide additional richness to the quantitative data.

## Results

**Fig. 1:**
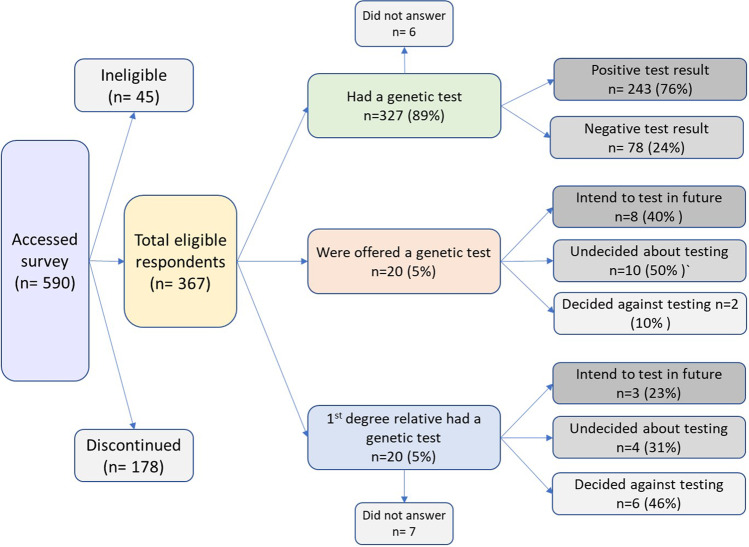
Characteristics of eligible respondents.

Overall, 367 individuals progressed through the eligibility questions and answered at least one substantive survey question, of 590 who initially accessed the survey (Fig. 1). The majority (89%; *n* = 327/367) had undertaken genetic testing (*genetic testers*), and of those who answered, 76% (*n* = 243/321) received a positive test result. Demographic characteristics of the respondents are summarised in Table 1. Demographic questions were answered at the end of the survey, and not all respondents answered all questions. Thus, *n* values are provided for each result reported. A range of genetic conditions was represented, with ~12–15% of respondents reporting testing associated with each of HBOC, cardiovascular disease, Lynch syndrome, mitochondrial disease, and haemochromatosis. Almost 30% (*n* = 94/326) reported having genetic testing after the moratorium came into effect on 1 July 2019.

**Table 1:** Demographic characteristics of respondents.

Demographic characteristics	*n*	%
**Sex (*n*** = **300)**		
Female	205	68.3
Male	93	31.0
Other/prefer not to say	2	0.7
**Age Group (*n*** = **298)**		
18–39	66	22.1
40–64	154	51.7
65 +	78	26.2
**Timing of test (*n*** = **326)**		
Before 1 July 2019	232	71.2
On or after 1 July 2019	94	28.8
**State/Territory (*n*** = **301)**		
Australian Capital Territory	14	4.7
New South Wales	75	24.9
Northern Territory	1	0.3
Queensland	64	21.3
South Australia	31	10.3
Tasmania	9	3.0
Victoria	74	24.6
Western Australia	33	11.0
**Highest level of education attained (*n*** = **300)**		
Some high school	26	8.7
Grade 12 equivalent/TAFE	76	25.3
Undergraduate qualification	76	25.3
Post-graduate qualification	111	37.0
Prefer not to say	11	3.7
**Conditions represented (*n*** = **367)**		
Lynch syndrome (bowel/uterine/other cancer) genes	58	15.8
Inherited cardiovascular disorder genes	57	15.5
Genes related to mitochondrial disease	55	15.0
Hereditary breast/ovarian cancer genes	53	14.4
Haemochromatosis	44	12.0
Genes related to neurodegenerative disease	17	4.6
Genes related to kidney disease	15	4.1
Peutz-Jeghers syndrome	4	1.1
Other	45	12.3
Don’t know	19	5.2

### Knowledge and awareness

Most respondents (74%; *n* = 256/348) reported not knowing whether Australian life insurance companies are legally allowed to use genetic test results in underwriting, and 9% incorrectly believed they are not allowed to (Table 2). Further, 84% (*n* = 286/340) had never heard of the FSC moratorium.

**Table 2:** Awareness of moratorium and opinions about regulation.

Question	Answer options	%	*n*
**Do you know whether Australian life insurance companies are legally allowed to use applicants’ genetic test results to decline an application, restrict cover or increase the cost of premiums?** **(*n*** = **348)**	They are allowed to	17.1	60
	They are not allowed to	9.2	32
	I am unsure	73.6	256
*[after the moratorium agreement is described]* **Have you heard about this agreement (called a moratorium)?** **(*n*** = **340)**	No	84.1	286
	Yes, I heard about this through the team that organised my genetic test	5.0	17
	Yes, I heard about this elsewhere	10.9	37
*[if yes]*, **To what extent did the agreement described above (the moratorium) influence your decision whether to have a genetic test?** **(*n*** = **54)**	It did not have any influence	77.8	42
	It had moderate influence	9.3	5
	It had significant influence	13.0	7
**Do you think life insurance companies should be allowed to use applicants’ genetic test results to decline an application, restrict cover or increase the cost of premiums?** **(*n*** = **350)**	Yes	4.0	14
	No	82.3	288
	Unsure	13.7	48
**In your opinion, what amount of life insurance cover (death cover) should applicants be allowed to apply for without having to disclose their genetic results?** **(*n*** = **341)**	No cover	0.9	3
	$250,000	3.2	11
	$500,000	11.7	40
	$1,000,000	17.9	61
	Unlimited cover	42.5	145
	Unsure	23.8	81
**How much do you agree/disagree with the following statement?** **The Australian government should introduce legislation (which is made and enforced by government) to regulate life insurers’ use of genetic test results** **(*n*** = **340)**	Strongly agree	62.4	212
	Agree	25.3	86
	Neither agree nor disagree	5.6	19
	Disagree	1.2	4
	Strongly disagree	1.5	5
	Can’t choose	4.1	14

### Use of genetic test results and regulation of insurers

**Fig. 2:**
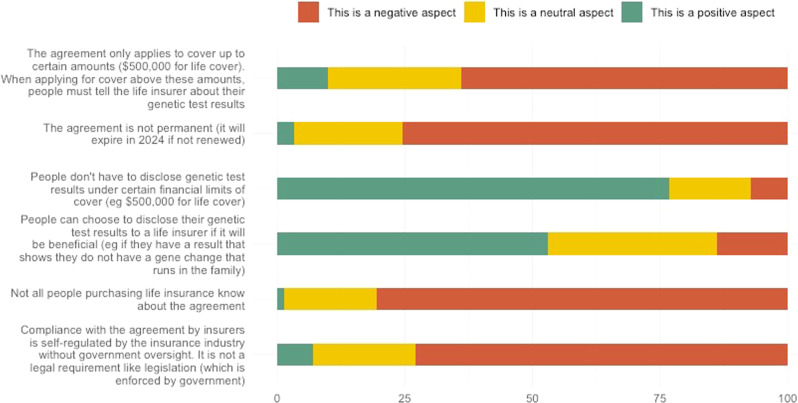
Respondents’ rating of aspects of the FSC moratorium.

A small number of respondents (4%; *n* = 14/350) said life insurance companies **should** be allowed to use genetic test results to decline an application, restrict cover or increase the cost of premiums. However, the majority (82%; *n*-288/350) said life insurance companies **should not** be allowed to (Table 2). Further, 73% (*n* = 219/300) rated the fact that *compliance with the agreement is self-regulated by the insurance industry without government oversight* as a negative aspect of the FSC moratorium, and only 7% rated it positive (Fig. 2 and Table S2). The fact that *the agreement is not permanent* was rated as a negative aspect by 76% (228/302) of respondents (only 3% rated it positive). When asked about regulation of the use of genetic test results in life insurance underwriting, 88% of respondents agreed or strongly agreed that government should introduce legislation (*n* = 298/340); only 3% (9/340) disagreed (Table 2).

### Financial limits of the FSC moratorium

Overall, 77% of respondents (*n* = 234/305) rated the fact that *people don’t have to disclose genetic test results under certain financial limits* as a positive aspect of the FSC moratorium (Fig. 2; Table S2). When asked about what amount of life insurance cover applicants should be allowed to apply for without being required to disclose their genetic results, only 16% (*n* = 54/341)) considered $500,000 or less was appropriate (Table 2). The majority (66%; *n* = 226/341) considered the amount of cover should be ≥ $1 million, with 64% of those (*n* = 145/226) stating the amount should be unlimited. One respondent described the FSC moratorium in free-text comments as a “tokenistic offering” by insurance companies, explaining, “*the vast majority of people applying for these insurances will have mortgages above that value. It does not cover basic needs*”.

### Access to insurance and the FSC moratorium’s influence on decision-making about genetic testing

Table 3 sets out findings relating to what type of life insurance cover respondents hold, when they obtained that cover, and difficulties with accessing cover. We asked respondents to distinguish between cover held within superannuation (either basic cover or extended cover) or outside superannuation, and obtained before or after the genetic test was undertaken. In Australia, superannuation refers to compulsory employer contributions to employees’ retirement funds. Superannuation funds generally offer a low level of cover for life insurance products without undertaking risk assessment (basic cover), but increasing this cover (extended cover) usually requires risk assessment. The amount of cover offered under “basic cover” varies between superannuation companies. In 2017, the median level of life insurance cover held by working Australians was estimated to be A$143,000, most of which was held through superannuation accounts [37].

**Table 3:** Life insurance cover held by respondents and difficulties accessing cover.

Do you hold the following types of cover?			%	*n*
**Life insurance (death cover)** **(*n*** = **211)**	**Yes** 46.4% (*n* = 98)	Basic cover through superannuation	38.0	35/92
		Extended cover through superannuation	22.8	21/92
		Cover outside of super (obtained before genetic test)	29.3	27/92
		Cover outside of super (obtained after genetic test)	9.8	9/92
	**No cover**		**48.3**	**102**
	Unsure		3.3	7
	Prefer not to say		1.9	4
**Total and permanent disability (TPD) cover (*n*** = **204)**	**Yes** 41.7% (*n* = 85)	Basic cover through superannuation	50.0	40/80
		Extended cover through superannuation	23.8	19/80
		Cover outside of super (obtained before genetic test)	18.8	15/80
		Cover outside of super (obtained after genetic test)	17.5	6/80
	**No cover**		**51.5**	**105**
	Unsure		4.9	10
	Prefer not to say		2.0	4
**Income protection/ salary continuance cover** **(*n*** = **196)**	**Yes** 33.2% (*n* = 65)	Basic cover through superannuation	47.5	29/61
		Extended cover through superannuation	21.3	13/61
		Cover outside of super (obtained before genetic test)	24.6	15/61
		Cover outside of super (obtained after genetic test)	6.6	4/61
	**No cover**		**63.3**	**124**
	Unsure		2.0	4
	Prefer not to say		1.5	3
**Trauma and/ or critical illness cover** **(*n*** = **193)**	**Yes** 21.2% (*n* = 41)	Basic cover through superannuation	29.7	11/37
		Extended cover through superannuation	16.2	6/37
		Cover outside of super (obtained before genetic test)	45.9	17/37
		Cover outside of super (obtained after genetic test)	8.1	3/37
	**No cover**		**67.9**	**131**
	Unsure		9.3	18
	Prefer not to say		1.6	3
**Have you ever had difficulty obtaining life (death cover), TPD, income protection or trauma/critical illness cover, based on your genetic test results? (*n*** = **284)**	Yes* 19.0% (*n* = 53)	Yes, I have had cover denied	8.1	23
		Yes, I have had a financial adviser tell me that I would not be able to get insurance	4.9	14
		Yes, I have had an increased premium applied	4.6	13
		Yes, I have had certain conditions placed on my cover	6.7	19
	No difficulties		35.9	102
	I have never tried to apply for, or made enquiries about, life insurance products		46.8	133
	*[if yes to never trying to apply]* **Did concerns about genetic discrimination influence your decision not to apply for life insurance products? (*n*** = **131)**	This did not have any influence	74.0	97
		This had moderate influence	11.5	15
		This had significant influence	15.4	19

Bold values show individuals with no cover.

Across each category of cover (death, total and permanent disability (TPD), income protection, and trauma/critical illness) around half of respondents reported that they had no cover. Overall, 42% who answered (*n* = 89/212) reported having **no** cover in any category. Of those who had insurance and reported their type of cover, most reported already having the cover in place before having genetic testing, or only obtaining basic cover within superannuation (70%; *n* = 77/110). Only 11% (*n* = 12/110) of those who reported having insurance obtained cover (other than basic cover within superannuation) after their genetic test.

**Fig. 3:**
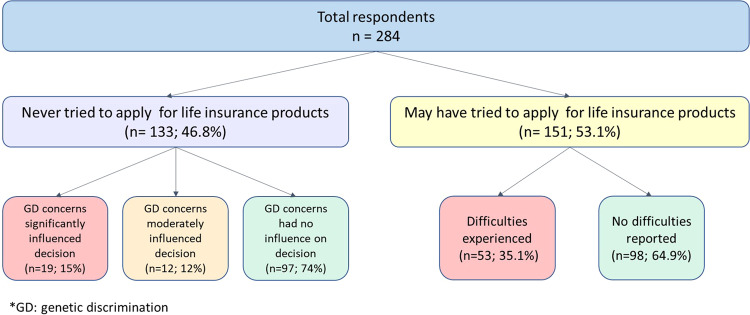
Decision-making about and difficulties experienced in applying for life insurance products.

Of 284 respondents, almost half (*n* = 133) reported they had never tried to apply for, or made enquiries about, life insurance products (Fig. 3). Of those, over a quarter (26%; *n* = 34/131) said genetic discrimination concerns had a moderate or significant influence on their decision not to apply for life insurance. Of those who may have tried to apply for life insurance products (ie they did not report that they had never tried to apply), over a third (*n* = 53/151) reported difficulties, including insurers rejecting applications; financial advisers telling respondents that their applications would be rejected; and insurers placing conditions on insurance policies or charging higher premiums. Types of insurance affected (more than one answer could be selected) were death cover (*n* = 38/51), TPD cover (*n* = 21/51), income protection (*n* = 22/51), and trauma/critical illness cover (*n* = 12/51). Of those who answered, 24% (*n* = 12/51) reported this difficulty happening after the introduction of the FSC moratorium on 1 July 2019. Details of those twelve are provided in Supplementary Table S3.

Some respondents reported discrimination even after taking preventive measures, exemplified by “Shona” (a pseudonym), a 43 year old woman with a *BRCA2* variant and family but no personal history of cancer (Table S3). Despite having her ovaries and fallopian tubes removed, and regular intensive breast imaging (mammogram/MRI/ultrasound), she was denied life insurance (death cover) outright with no justification or explanation from the insurer.

When those who had heard about the FSC moratorium were asked to what extent it influenced their decision whether to have a genetic test, the majority stated that it did not have an influence (78%; *n* = 42/54), with the remaining 22% saying it had a moderate/significant influence (Table 2). Half of the respondents who had decided against, or had not yet had, genetic testing reported that concerns about life insurance had a moderate/significant effect on their decision making (50%; *n* = 7/14). One respondent reported in free text that he decided not to have testing because of life insurance issues “*such as exclusions or increased premiums that may arise because of the test*”. He said, “*at the moment it is better to be in the dark*”. Two individuals who were undecided about having testing also provided more detail in free text comments – one mentioned the uncertainty about whether the moratorium would continue past 2024, and the other stated they wanted to discuss the life insurance situation with family members before deciding about testing.

Ten individuals provided free-text comments at the end of the survey – seven reported having positive tests for pathogenic variants, two were undecided about testing, and one was intending to have testing. Of those who had positive tests, two mentioned frustration that insurers chose to discriminate rather than encouraging individuals to be proactive or take risk-reducing measures. One participant stated, “*if I’m aware of my genetic condition and keep up with my screening, I don’t think I should be discriminated against. I should be rewarded for being proactive*”.

Six respondents outlined concerns about future life insurance discrimination for people who have genetic testing or their family members. One participant stated, “*I continue to be worried for my relatives. It has caused family members to hold off on very important gene testing*”. One respondent with a family history of Lynch syndrome reported that they had been intending to have testing, and although they had trauma cover already, they had not had testing yet because they wanted to obtain new cover with a different insurer before proceeding with testing. They reported that an underwriter informed them that the insurer would not cover them without being told about their genetic test results once they had them, and so this process was continuing to hold up their decision to proceed with testing.

## Discussion

Overwhelmingly, our findings demonstrate ongoing consumer concerns about genetic discrimination in life insurance, by people having or considering genetic testing. They also show low awareness about the legality of this practice, or the existence of the FSC moratorium. Our study presents further evidence of ongoing consumer difficulties accessing life insurance products, despite the FSC moratorium being in place. Most respondents (88%) had a strong view that government regulation is required in this area.

Respondents’ awareness regarding the use of genetic test results by life insurers was limited, with less than 20% being aware that the practice is legal, or that the FSC moratorium exists. This lack of awareness regarding the legal status of genetic discrimination is reflected internationally [38, 39], and was accompanied by an overwhelming view that using genetic test results in life insurance should not be allowed. Further, when information about the terms of the moratorium were provided (see Supplementary file S1, p14), it became clear that consumers did not consider it to be an adequate mechanism for regulating this issue, with a large majority rating the moratorium’s temporary nature, industry self-regulation, and low financial limits as negative aspects. Respondents consider that the moratorium’s limits are too low, highlighted by comments that the limit is a “tokenistic offering” by the insurance industry, which “does not cover basic needs”.

Our findings suggest that the moratorium has provided some modest benefit to consumers, which should be acknowledged. This includes some aspects rated as positive by the majority of participants, such as the fact that *people don’t have to disclose genetic test results under certain financial limits* and *people can choose to disclose their genetic tests to a life insurer if it will be beneficial*. A small minority of those who had heard of the moratorium (13%) stated that it had a significant influence on their decision to have genetic testing, which is a positive outcome. However, the majority (78%) reported it having no influence on their decision-making about having testing. There is a paucity of international research regarding the impact of moratoria or other regulations on decision-making regarding genetic testing. However, a US study showed that participants’ hypothetical interest in participating in genetic research decreased when they were provided with more detailed information about the limitations in protection offered by US genetic non-discrimination legislation [40].

Further, concerns about genetic discrimination are still influencing consumer decision-making regarding genetic testing in Australia, both with regards to having genetic testing and deciding whether to try to access life insurance products. Half of the respondents who had not had testing, and over a quarter of those who had not tried to apply for life insurance products, reported life insurance concerns as having a moderate or significant effect on their decision-making. Similarly, >two-thirds of US study participants asked about interest in genetic testing (in four states where genetic discrimination legislation does not protect life insurance), had concerns about use of genetic test results by life insurance companies [41].

Of particular concern were reports that consumers continue to have difficulty accessing life insurance products, and still experience discrimination based on genetic test results, even after the introduction of the FSC moratorium. Several respondents commented on the failure of insurers to consider preventive measures, and some respondents reported experiencing discrimination even after taking preventive measures. A 1998 study conducted in the UK before the introduction of its moratorium similarly reported instances of unjustified genetic discrimination of individuals who did not present adverse actuarial risk [27]. No studies have reported whether this unjustified discrimination continued after the introduction of the UK moratorium. The ongoing failure to consider preventive measures is not only frustrating for proactive patients seeking to obtain insurance coverage and contrary to the requirements under section 46 of the DDA, but also inconsistent with the life insurance industry’s commitment to evidence-based actuarial practice. Taking breast cancer as an example, survival is very high for women whose breast cancer is detected early. The five-year survival of women with breast cancer is now at least 92% [42], almost as high as for those without breast cancer (98% relative survival rate) for early-stage cancers ≤ 10 mm [43]. In *BRCA1/2* carriers, annual imaging significantly reduces the incidence of later stage breast cancers [44]. Thus, for women like our example, “Shona”, who had preventive surgery and is having regular intensive breast screening, their likelihood of survival *even if they do develop breast cancer* is very high. However, our findings show Australian life insurance companies still refuse life cover to some such women, on the basis of their genetic test results. Comparatively, if Shona was not aware of her increased genetic predisposition, and did not take preventive steps, her likelihood both of developing cancer and dying from that cancer would be significantly higher.

Our findings also show that some people at risk of having genetic predisposition to medically-actionable conditions continue to choose not to have genetic testing because of insurance discrimination fears, despite the introduction of the FSC moratorium. This finding is consistent with reports from health professionals who discuss clinically-indicated genetic testing with at-risk individuals [23], who have reported that people continue to delay and decline testing because of insurance fears. Enabling at-risk individuals to have genetic testing without fear of discrimination will increase risk prevention and ultimately decrease the likelihood of insurance claims [45]. This means anti-discrimination regulation is also in the interests of insurers, despite their frequent opposition, and should be supported.

Many respondents reported having no cover across any life insurance products, including a significant number in the 40–64 year old age group. Anecdotal estimates regarding how many Australians hold life insurance vary, but accurate estimates are difficult to obtain. In 2015, the Australian Securities and Investment Commission reported that there were 21.9 million active policies for life insurance products [46]. Many of those (14 million) were group insurance products within superannuation (and it is likely that some individuals with multiple employers may have multiple superannuation accounts and several low-level insurance product policies).

In our study, the majority of those who reported having cover were fortunate to have obtained it before having genetic testing, or only had basic levels of cover through their superannuation. Although having a low level of cover is better than no cover, the median default level of cover within superannuation funds does not adequately cover Australians’ financial needs, especially parents with young children [47]. Very few individuals in this study had successfully obtained cover (outside of basic superannuation cover) *after* receiving their genetic test results. Some individuals reported that although they had not been declined formally by an insurer, their financial adviser told them that they wouldn’t be able to obtain cover, highlighting the critical role played by financial advisers in managing access to insurance for individuals with genetic test results.

Our current study builds upon our previous findings from a survey conducted pre-moratorium [31], in which many individuals reported having difficulties accessing life insurance after genetic testing. In the previous survey, numerous individuals reported genetic discrimination by life insurers even after taking preventive measures for hereditary cancer predisposition. Unfortunately, such instances are unlikely to be mitigated by the introduction of the FSC moratorium, which already requires insurers to consider preventive measures but is not enforceable. Only enforceable regulation by government can meaningfully impact insurers’ use of genetic information. The current survey was circulated to consumers less than 18 months after the FSC’s introduction, which may have limited the number of new instances of discrimination which it captured. However, this research demonstrates that such discrimination is still occurring and will likely continue to occur in the absence of enforceable regulation.

Limitations of our study include responder bias – it is likely that those who are more interested in this topic would have chosen to complete the survey. However, conversely, individuals who are strongly against testing because of discrimination concerns often won’t engage with genetics services or be involved in research, meaning that our survey may also have failed to capture many individuals with strong discrimination concerns. Further, because the survey could be completed anonymously, not all respondents who reported experiences of discrimination could be contacted for more information. A separate qualitative study is now underway, which will interview respondents who have agreed to be contacted. A separate survey of the general public has also been undertaken, which will elucidate any relevant differences between the views of the patient population reported in this paper from those of the general Australian public. Further research is required to document the views of individuals who have decided against genetic testing (decliners), who are difficult to recruit into research studies.

Our study findings demonstrate that, despite the introduction of the FSC moratorium, fears of genetic discrimination persist in Australia, and continue to deter some individuals from having genetic testing. This suggests that the FSC moratorium is not adequately easing insurance discrimination fears for Australian consumers considering genetic testing. Consumers continue to experience genetic discrimination in life insurance, and overwhelmingly believe that life insurers should not be allowed to use genetic test results in underwriting, and that the Australian government should introduce legislation to regulate this area. This study adds to the growing body of evidence that must be considered by the Australian government in determining whether further regulation is now required. Future research should gather views of the Australian public more broadly about this issue. Our findings to date strongly suggest that the current FSC moratorium is not providing Australian consumers with sufficient reassurance and protection, and that the government should consider the implementation of legislation prohibiting the use of genetic test results in life insurance underwriting.

### Supplementary information

Supplementary file S1

Supplementary Table S2

Supplementary Table S3

## Data Availability

Numerous data are made available via supplementary materials. Additional data can be made available on reasonable request.
