# Correction: Community concerns about genetic discrimination in life insurance persist in Australia: A survey of consumers offered genetic testing

**DOI:** 10.1038/s41431-023-01391-z

**Published:** 2023-05-22

**Authors:** Jane Tiller, Andrew Bakshi, Grace Dowling, Louise Keogh, Aideen McInerney-Leo, Kristine Barlow-Stewart, Tiffany Boughtwood, Penny Gleeson, Martin B. Delatycki, Ingrid Winship, Margaret Otlowski, Paul Lacaze

**Affiliations:** 1 Public Health Genomics, School of Public Health and Preventive Medicine, Monash University, Melbourne, Australia; 2 Murdoch Children’s Research Institute, Parkville, Australia; 3 Australian Genomics, Melbourne, Australia; 4 Centre for Health Equity, Melbourne School of Population and Global Health, The University of Melbourne, Melbourne, Australia; 5 The University of Queensland Diamantina Institute, University of Queensland, Dermatology Research Centre, Brisbane, Australia; 6 Northern Clinical School, Faculty of Medicine and Health, University of Sydney, Sydney, Australia; 7 Deakin Law School, Melbourne, Australia; 8 Victorian Clinical Genetics Services, Parkville, Australia; 9 Department of Medicine, the University of Melbourne, Melbourne, Australia; 10 Genomic Medicine and Family Cancer Clinic, Royal Melbourne Hospital, Parkville, Australia; 11 Faculty of Law and Centre for Law and Genetics, University of Tasmania, Hobart, Australia

**Keywords:** Genetic counselling, Medical ethics, Genetics, Ethics, Clinical genetics

Correction to: *European Journal of Human Genetics* 10.1038/s41431-023-01373-1, published online 11 May 2023

In the sentence beginning ‘When asked about regulation of the use of genetic test results…’ in this article, the value ‘91%’ should have been ‘88%’.

The original article has been corrected.

