# Supplementary material for: Community concerns about genetic discrimination in life insurance persist in Australia: A survey of consumers offered genetic testing
Source: Eur J Hum Genet. 2023 May 11;32(3):286–94. doi: 10.1038/s41431-023-01373-1 (PMC10923945; doi:10.1038/s41431-023-01373-1)
Supplement: Supplementary file 1 — Supplementary file S1 [file 41431_2023_1373_MOESM1_ESM.pdf]

Genetic testing and life insurance

The use of genetic information is concerning to many, especially in the context of life insurance. Our research aims to understand the views and experiences of people who have had genetic testing, or are eligible for testing, for gene changes which increase the risk of developing disease.

Please download and read the Explanatory Statement below and continue with the eligibility questions if you would like to participate.

[Attachment: "Explanatory statement consumer survey.pdf"]

Are you over 18 years of age?	◯ Yes<br>◯ No
Unfortunately this survey is restricted to individuals over the age of 18<br><br>Thank you for your interest in our study.	◯ OK
Do you live in Australia?	◯ Yes<br>◯ No
Unfortunately this survey is restricted to individuals living in Australia.<br><br>If you live outside of Australia and would like to share your story with an international team of researchers interested to hear about genetic discrimination, you may click here to visit the Genetic Discrimination Observatory	◯ OK

This survey is for Australians who have had, or are eligible for, a genetic test for a gene change that increases the chance of developing disease (either before or after developing symptoms of disease).

***This does not include pre-conception carrier screening or prenatal testing, but does include predictive genetic testing**

Have you or your relative been offered a genetic test as described above?<br>(if more than one, choose the one that best applies)	◯ Yes - I have had such a genetic test<br>◯ Yes - I have been offered such a test but have not taken the test at this time<br>◯ Yes - my first degree relative* has had such a genetic test but I have not had a test<br>◯ No<br>(*a first-degree blood relative is a sibling, parent or child)
Unfortunately this survey is restricted to individuals who have had, or are eligible for, a genetic test for a gene change that increases the chance of developing disease (either before or after developing symptoms of disease).<br><br>Thank you for your interest in our study.	◯ OK
What is the relative's relationship to you? (if multiple relatives have had a test, select the option that applies to your oldest relative)<br><br>They are my:	◯ mother or father<br>◯ brother or sister<br>◯ daughter or son

When you click "submit", you will be taken to the main survey questions :)

Genetic testing and life insurance survey

The following questions are about the genetic test [you_your_proxy]

Wording for 'you' and 'your relative'	◯ you have had<br>◯ you were offered<br>◯ your relative had
What was the genetic test [you_your_proxy]?	◯ Hereditary breast/ovarian cancer genes<br>◯ Lynch syndrome (bowel/uterine/other cancer) genes<br>◯ Inherited cardiovascular disorder genes<br>◯ Genes related to mitochondrial disease<br>◯ Genes related to neurodegenerative disease<br>◯ Other ______<br>◯ Don't know
Which gene?	◯ BRCA1<br>◯ BRCA2<br>◯ PALB2<br>◯ ATM<br>◯ CHEK2<br>◯ CDH1<br>◯ Other<br>◯ Unsure
Which gene?	◯ MLH1<br>◯ MSH2<br>◯ MSH6<br>◯ PMS2<br>◯ Other<br>◯ Unsure
Which gene?	◯ Inherited rhythm disorder genes<br>◯ Inherited cardiomyopathy genes<br>◯ Inherited high cholesterol genes<br>◯ Other<br>◯ Unsure
If other, please specify	__________________________________
Did you have your genetic test (your best recollection)	◯ Before 1 July 2019<br>◯ On or after 1 July 2019<br>(We ask this because of a change in regulation, which will be explained later in the survey)
What was the result of the test [you_your_proxy]?	◯ Positive (the genetic change tested for was detected)<br>◯ Negative (the genetic change tested for was not detected)
Have you thought about having a genetic test for the gene change found in your family member/s?	◯ I am intending to have a test in the future (optional comment): ______<br>◯ I have not decided whether I want to have a test (optional comment): ______<br>◯ I have decided not to have a test
What are your thoughts about having the genetic test that has been offered to you?	◯ I am intending to have a test in the future (optional comment): ______<br>◯ I have not decided whether I want to have a test (optional comment): ______<br>◯ I have decided not to have a test

We are interested to understand and support the decision-making of people who choose not to have a test - we are not trying to change your mind about your decision.<br><br>Can you tell us why you have chosen not to have a test?	__________________________________<br>(*optional text)
What effect (if any), did concerns about the use of genetic test results in life insurance underwriting have on your decision-making about having a genetic test?	◯ It did not have any effect on my decision<br>◯ It has had a moderate effect on my decision<br>◯ It has had a significant effect on my decision

The reason we ask the following questions is to try to understand better the experience of individuals with genetic changes that increase their chance of developing disease. You can skip any questions you feel uncomfortable answering.

Have you had one or more diagnoses of breast, ovarian and/or other cancer? [Select all that apply]	☐ Yes, breast cancer<br>☐ Yes, ovarian cancer<br>☐ Yes, other cancer ______<br>☐ No
When was the breast cancer diagnosed?	◯ before 1 July 2019<br>◯ on or after 1 July 2019
When was the ovarian cancer diagnosed?	◯ before 1 July 2019<br>◯ on or after 1 July 2019
When was the [specify] cancer diagnosed?	◯ before 1 July 2019<br>◯ on or after 1 July 2019
To your knowledge, have any of your first-degree relatives* had one or more diagnoses of breast and/or ovarian cancer?	☐ Yes, breast cancer<br>☐ Yes, ovarian cancer<br>☐ Yes, other cancer ______<br>☐ No<br>(*a first-degree blood relative is a sibling, parent or child)
When was the first breast cancer diagnosed?	◯ before 1 July 2019<br>◯ on or after 1 July 2019
When was the first ovarian cancer diagnosed?	◯ before 1 July 2019<br>◯ on or after 1 July 2019
When was the first [specify_2] cancer diagnosed?	◯ before 1 July 2019<br>◯ on or after 1 July 2019
Have you ever had risk-reducing surgery (this means surgery before any cancer in that location)?	☐ Unilateral mastectomy<br>☐ Bilateral mastectomy<br>☐ Removal of one ovary<br>☐ Removal of both ovaries<br>☐ Removal of fallopian tubes<br>☐ Hysterectomy<br>☐ Other ______<br>☐ No surgery
When did you have the removal of one ovary?	◯ before 1 July 2019<br>◯ on or after 1 July 2019

When did you have a unilateral mastectomy?
◯ before 1 July 2019
◯ on or after 1 July 2019

When did you have the removal of both ovaries?
◯ before 1 July 2019
◯ on or after 1 July 2019

When did you have the removal of your fallopian tubes?
◯ before 1 July 2019
◯ on or after 1 July 2019

When did you have a bilateral mastectomy?
◯ before 1 July 2019
◯ on or after 1 July 2019

When did you have a hysterectomy?
◯ before 1 July 2019
◯ on or after 1 July 2019

When did you have this [other_surgery] ?
◯ before 1 July 2019
◯ on or after 1 July 2019

Do you take any medication to prevent the development of cancer?
◯ Yes
◯ No

Please comment on the medication that you take
____

Do you have a screening programme of regular breast imaging (mammogram, MRI, ultrasound/other)?
◯ Yes
◯ No

What is your usual screening program?

	6-monthly or more frequently	Every 6-12 months	Every 1 - 2 years	Less than every 2 years	Never
Mammogram	◯	◯	◯	◯	◯
MRI	◯	◯	◯	◯	◯
Ultrasound	◯	◯	◯	◯	◯
other ______	◯	◯	◯	◯	◯

Have you had one or more diagnoses of cancer and/or precancerous cells? (Select all that apply)
☐ Bowel polyps
☐ Bowel (colon) cancer
☐ Endometrial (uterine or womb) cancer
☐ Ovarian cancer
☐ Pancreatic cancer
☐ Other cancer ______
☐ No cancer diagnosis or precancerous cells

When were the bowel polyps first diagnosed?
◯ before 1 July 2019
◯ on or after 1 July 2019

When was the colon cancer diagnosed?
◯ before 1 July 2019
◯ on or after 1 July 2019

When was the endometrial cancer diagnosed?
◯ before 1 July 2019
◯ on or after 1 July 2019

When was the ovarian cancer diagnosed?
◯ before 1 July 2019
◯ on or after 1 July 2019

When was the pancreatic cancer diagnosed?	◯ before 1 July 2019<br>◯ on or after 1 July 2019
When was the [other4] cancer diagnosed?	◯ before 1 July 2019<br>◯ on or after 1 July 2019
Have you ever had risk-reducing surgery (this means surgery before any cancer in that location)?	☐ Hysterectomy (uterus/womb)<br>☐ Hysterectomy and bilateral salinpgo-oopherectomy (uterus/womb and ovaries)<br>☐ Other ______<br>☐ No surgery
When did you have the hysterectomy?	◯ before 1 July 2019<br>◯ on or after 1 July 2019
When did you have the hysterectomy/oopherectomy?	◯ before 1 July 2019<br>◯ on or after 1 July 2019
When did you have the [other6]?	◯ before 1 July 2019<br>◯ on or after 1 July 2019
To your knowledge, have any of your first-degree relatives* had one or more diagnoses of cancer and/or precancerous cells? (Select all that apply)	☐ Bowel polyps<br>☐ Bowel (colon) cancer<br>☐ Other cancer ______<br>☐ No<br>☐ I don't know<br>(*a first-degree blood relative is a sibling, parent or child)
When was the first bowel cancer diagnosed?	◯ before 1 July 2019<br>◯ on or after 1 July 2019
When were the bowel polyps first diagnosed?	◯ before 1 July 2019<br>◯ on or after 1 July 2019
When was the first [other5] cancer diagnosed?	◯ before 1 July 2019<br>◯ on or after 1 July 2019
Do you have a screening programme involving any of the following (Select all that apply)	☐ Colonoscopy<br>☐ Endoscopy<br>☐ Radiological tests (ultrasound)<br>☐ Blood tests (CEA, CA 19, CA 125, PSA)<br>☐ Skin check<br>☐ Other ______<br>☐ No
Is your colonoscopy screening	◯ 6-monthly or more frequently<br>◯ Every 6-12 months<br>◯ Every 12 months - 2 years<br>◯ Less than every 2 years<br>◯ Other ______
Is your endoscopy screening:	◯ 6-monthly or more frequently<br>◯ Every 6-12 months<br>◯ Every 12 months - 2 years<br>◯ Less than every 2 years<br>◯ Other ______

Is your radiological screening:	◯ 6-monthly or more frequently<br>◯ Every 6-12 months<br>◯ Every 12 months - 2 years<br>◯ Less than every 2 years<br>◯ Other ______
Are your blood tests:	◯ 6-monthly or more frequently<br>◯ Every 6-12 months<br>◯ Every 12 months - 2 years<br>◯ Less than every 2 years<br>◯ Other ______
Is your skin check	◯ 6-monthly or more frequently<br>◯ Every 6-12 months<br>◯ Every 12 months - 2 years<br>◯ Less than every 2 years<br>◯ Other ______
Is your [lynch_other_screening] screening	◯ 6-monthly or more frequently<br>◯ Every 6-12 months<br>◯ Every 12 months - 2 years<br>◯ Less than every 2 years<br>◯ Other ______
Do you take any medication to prevent the development of cancer?	◯ Yes<br>◯ No
Please comment on the medication that you take	__________________________________
Do you have a clinical diagnosis of a genetic condition?	◯ Yes<br>◯ No
What condition is your genetic test associated with?	__________________________________
When were you diagnosed?	◯ before 1 July 2019<br>◯ on or after 1 July 2019
What is the name of the condition?	__________________________________
To your knowledge, have any of your first-degree relatives* had one or more diagnoses of a genetic condition associated with this genetic test?	◯ Yes<br>◯ No<br>◯ I don't know<br>(*a first-degree blood relative is a sibling, parent or child)
What is the name of the condition?	__________________________________
When was the condition first diagnosed in a relative?	◯ before 1 July 2019<br>◯ on or after 1 July 2019
Do you have any screening or regular testing done to look for signs and symptoms of the genetic condition?	◯ Yes<br>◯ No

Please comment on the screening/testing you have done and how frequently this is done	__________________________________
Have you had any medical procedure/s to treat or reduce your risk of developing the genetic condition or to treat symptoms of the condition?	◯ Yes<br>◯ No
Please comment on the treatment or risk-reducing measures	__________________________________
Do you take any medication to slow the progress of, prevent or treat the symptoms of the genetic condition ?	◯ Yes<br>◯ No
Please comment on the medication that you take	____________________________
Have you taken any other measures for prevention, early detection or treatment of the genetic condition?	◯ Yes<br>◯ No
Please tell us some more about this	__________________________________

These questions ask what you already know about the use of genetic test results by life insurers in Australia

Do you know whether Australian life insurance companies are legally allowed to use applicants' genetic test results to decline an application, restrict cover or increase the cost of premiums?	◯ They are allowed to<br>◯ They are not allowed to<br>◯ I am unsure
Do you think life insurance companies should be allowed to use applicants' genetic test results to decline an application, restrict cover or increase the cost of premiums?	◯ Yes<br>◯ No<br>◯ Unsure

From 1 July 2019 to 30 June 2024, Australian life insurance companies have agreed that you don't have to disclose your genetic test results when applying for a policy that is under a certain amount.

Have you heard about this agreement (called a moratorium)?	◯ Yes, I heard about this through the team that organised my genetic test<br>◯ Yes, I heard about this elsewhere ______<br>◯ No
To what extent did the agreement described above (the moratorium) influence your decision whether to have a genetic test?	◯ It did not have any influence<br>◯ It had moderate influence<br>◯ It had significant influence
In your opinion, what amount of life insurance cover (death cover) should applicants be allowed to apply for without having to disclose their genetic results?	◯ $250,000<br>◯ $500,000<br>◯ $1,000,000<br>◯ Unlimited cover<br>◯ No cover<br>◯ Unsure
How much do you agree/disagree with the following statement?<br><br>The Australian government should introduce legislation (which is made and enforced by government) to regulate life insurers' use of genetic test results.	◯ Strongly agree<br>◯ Agree<br>◯ Neither agree nor disagree<br>◯ Disagree<br>◯ Strongly disagree<br>◯ Can't choose

If you feel comfortable telling us confidentially, could you select which of the following life insurance products (if any) you have currently?

	Yes	No cover	Unsure	Prefer not to say
Total and permanent disability (TPD) cover	◯	◯	◯	◯
Life insurance (death cover)	◯	◯	◯	◯
Income protection/salary continuance cover	◯	◯	◯	◯
Trauma and/or critical illness cover	◯	◯	◯	◯

Is your total and permanent disability (TPD) cover

◯ basic cover through super-annuation
◯ extended cover through super-annuation
◯ cover outside of super-annuation (obtained before genetic test)
◯ cover outside of super-annuation (obtained after genetic test)

Is your life insurance (death cover)

◯ basic cover through super-annuation
◯ extended cover through super-annuation
◯ cover outside of super-annuation (obtained before genetic test)
◯ cover outside of super-annuation (obtained after genetic test)

Is your income protection/salary continuance cover

◯ basic cover through super-annuation
◯ extended cover through super-annuation
◯ cover outside of super-annuation (obtained before genetic test)
◯ cover outside of super-annuation (obtained after genetic test)

Is your trauma and/or critical illness cover

◯ basic cover through super-annuation
◯ extended cover through super-annuation
◯ cover outside of super-annuation (obtained before genetic test)
◯ cover outside of super-annuation (obtained after genetic test)

Have you ever had difficulty obtaining life (death cover), TPD, income protection or trauma/critical illness cover, based on your genetic test results?

☐ Yes, I have had cover denied
☐ Yes, I have had a financial adviser tell me that I would not be able to get insurance
☐ Yes, I have had an increased premium applied
☐ Yes, I have had certain conditions placed on my cover (optional comment) ______
☐ No difficulties
☐ I have never tried to apply for, or made enquiries about, life insurance products

Did concerns about genetic discrimination influence your decision not to apply for life insurance products?

◯ This did not have any influence
◯ This had moderate influence
◯ This had significant influence

If you are comfortable telling us confidentially, what level of cover were you applying for (Select all that apply)	☐ Life (death cover) insurance<br>☐ Total and permanent disability (TPD) cover<br>☐ Income protection/salary continuance cover<br>☐ Trauma/critical illness cover<br>☐ I would prefer not to say
Were you applying for	◯ life insurance within superannuation?<br>◯ life insurance outside of superannuation?
What is the life (death) cover amount?	◯ Up to and including $500,000<br>◯ More than $500,000
What is the TPD cover amount?	◯ Up to and including $500,000<br>◯ More than $500,000
What is the income protection/ salary continuance cover amount?	◯ Up to and including $4000/month<br>◯ More than $4000/month
What is the trauma/critical illness cover amount?	◯ Up to and including $200,000<br>◯ More than $200,000
What information (if any) did the insurer/s provide to you to justify or explain the decision? If you remember the name of the insurer/s and/or have copies of any correspondence with the insurer/s that you are happy to provide, please let us know.	______________________________
When did this happen (your best recollection)	◯ Before 1 July 2019<br>◯ On or after 1 July 2019

The moratorium (ban) described previously is written and enforced by the life insurance industry, not by the government. From 1 July 2019 to 30 June 2024 (if not extended), life insurers have agreed not to ask for or use applicants' genetic test results when underwriting policies worth up to:

- **$500,000 for life cover,**
- **$200,000 for trauma/critical illness cover, and**
- **$4000/month for income protection.**

For policies worth over this amount, life insurers will be able to use genetic test results when underwriting.

For more information about the moratorium you can click here (opens in new window)

Below we have listed some key aspects of the agreement described above, that have been set by the insurance industry. Please indicate your opinion about each:

	This is a positive aspect	This is a neutral aspect	This is a negative aspect
People don't have to disclose genetic test results under certain financial limits of cover (eg $500,000 for life cover)	○	○	○
Compliance with the agreement by insurers is self-regulated by the insurance industry without government oversight. It is not a legal requirement like legislation (which is enforced by government)	○	○	○
The agreement is not permanent (it will expire in 2024 if not renewed)	○	○	○
The agreement only applies to cover up to certain amounts ($500,000 for life cover). When applying for cover above these amounts, people must tell the life insurer about their genetic test results	○	○	○

People can choose to disclose their genetic test results to a life insurer if it will be beneficial (eg if they have a result that shows they do not have a gene change that runs in the family)	◯	◯	◯
Not all people purchasing life insurance know about the agreement	◯	◯	◯

Given the life insurance and genetics moratorium is now in place, have you tried again to apply for, or are you considering applying for life, TPD, income protection or trauma/critical illness cover, after 1 July 2019?

◯ Yes, I have re-applied for new cover or tried to change my existing level of cover after 1 July 2019
◯ Yes, I am considering re-applying for new cover/ changing my existing level of cover in the future
◯ I haven't re-applied, because the moratorium doesn't make a difference to my ability to obtain cover
◯ No (other reason) ______

What was the outcome?

◯ Successful - I obtained the cover I applied for without an increase in premium or restrictions
◯ I was denied cover again on the basis of my genetic test results
◯ An increased premium was applied on the basis of my genetic test results
◯ Other ______

Did you try to challenge or appeal the insurer's decision?

◯ Yes, Optional comment: ______
◯ No

What was the outcome of the appeal?

____

Can you tell us why not?

____

Are you willing to speak confidentially with a member of the research team about your views or experiences, or about your plans to re-apply for life insurance?

◯ Yes
◯ No

Please provide your name (first name only is fine), email address and/or contact number. We will only use your personal details to contact you for this purpose and will not share any of your details outside of the research team without your consent.

Name: Email: Contact Phone Number:

______ ______ ______

Do you have any further comments to make about the insurance and genetics moratorium, genetic discrimination in life insurance, your experiences with accessing insurance after genetic testing, or any other related matters?

____

Final Questions

Which state/territory do you live in?	◯ Australian Capital Territory<br>◯ Queensland<br>◯ New South Wales<br>◯ Northern Territory<br>◯ South Australia<br>◯ Tasmania<br>◯ Victoria<br>◯ Western Australia<br>(*this is a required field)
What is your year of birth?	____________________________<br>(*this is a required field)
Gender	◯ Male<br>◯ Female<br>◯ Non-binary<br>◯ Other/prefer not to say<br>(*this is a required field)
Highest level of education attained:	◯ Some high school<br>◯ Grade 12 equivalent/TAFE qualification<br>◯ Undergraduate<br>◯ Post-graduate<br>◯ Prefer not to say<br>(*this is a required field)

Thank you for taking the time to complete the A-GLIMMER survey.

Please feel free to share the link with family members or other networks: https://redcap.link/aglimmer.consumer

Have a wonderful day.

Proxy tester

The following questions are about the genetic test [you_your_proxy]

123) Wording for 'you' and 'your relative'

- ◯ you have had
- ◯ you were offered
- ◯ your relative had

124) What was the genetic test [you_your_proxy] ?

- ◯ Hereditary breast/ovarian cancer genes
- ◯ Lynch Syndrome (colon/bowel cancer) genes
- ◯ Inherited cardiovascular disorder genes
- ◯ Genes related to mitochondrial disease
- ◯ Genes related to neurodegenerative disease
- ◯ Other {other_genes}
- ◯ Don't know
